# The relationship between teachers’ emotional intelligence and teaching for creativity: The mediating role of working engagement

**DOI:** 10.3389/fpsyg.2022.1014905

**Published:** 2022-12-22

**Authors:** Huili Su, Jingwei Zhang, Mingyue Xie, Ming Zhao

**Affiliations:** ^1^Faculty of Education, Northeast Normal University, Changchun, China; ^2^Ideological and Political Education Research Center, Northeast Normal University, Changchun, China

**Keywords:** emotional intelligence, teaching for creativity, working engagement, teachers, positive emotions

## Abstract

Teaching for creativity (TfC) has received increasing attention as an important way to cultivate students’ creative thinking and behaviors. The purpose of this study is to examine the mediating role of teachers’ work engagement (WE) on the relationship between their emotional intelligence (EI) and teaching for creativity. The study is a cross-sectional design. The sample of the study is 3,307 secondary school English teachers working in Jilin Province, China. The findings show that the teachers’ perceptions of emotional intelligence, work engagement and teaching for creativity are relatively high. The findings confirm the hypotheses. The results of structural equation modeling and bootstrapping show that teachers’ emotional intelligence is positively correlated with work engagement and teaching for creativity, and teachers’ work engagement mediates the relationship between emotional intelligence and teaching for creativity.

## 1. Introduction

In the knowledge economy era, creativity has become one of the most important qualities for today’s talent (Sawyer, 2006) and thus an imperative in curriculum and teaching reforms. Teaching for creativity (TfC) is a form of teaching and learning intended to support the nurturing of students’ creative thinking or behaviors (NACCCE, 1999; Jeffrey and Craft, 2004). In the research on teacher education, how to realize TfC has become a focus in recent years (Beghetto et al., 2014; Starko, 2017). Several studies have shown that creative skills are not only inherently related to learning (Jeffrey and Craft, 2004; Kaufman and Beghetto, 2009) but also related to teachers’ professional development, self-efficacy, and well-being (Sternberg, 2010; Chan and Yuen, 2014). Previous studies have explored the factors that predict creative teaching, including the external school environment (such as collaboration in teaching, school equipment and resources), teachers’ involvement in school decision-making (Collie et al., 2011; Bereczki and Kárpáti, 2018; Rubenstein et al., 2018) and internal personal factors related to teachers such as teacher enthusiasm, metacognition, creativity beliefs, and creative personality (Chan and Yuen, 2014; Huang et al., 2021). However, few studies have explored the impact of teachers’ emotional intelligence on teaching for creativity. Individual studies have investigated the relationship between teachers’ emotional intelligence and creativity, but the results are inconsistent (Pirkhaefi and Rafieyan, 2012; Ebrahimi et al., 2018; Awwad, 2022). In the field of teacher education, a number of studies have confirmed the positive impact of emotional intelligence on instructional performance. Teachers can be seen as emotional workers (Yin et al., 2019); their self-efficacy, teaching performance, burnout, job satisfaction, and teaching effectiveness have been found to be influenced by their emotions (Lavy and Eshet, 2018). English language instruction is a very emotional career, and the EI of English as a foreign language (EFL) educators significantly affects teachers’ health and learners’ education (Chang, 2013; Kang, 2022) and particularly affects the variety of teaching techniques and instructional imagination of EFL teachers (Shen, 2022). Therefore, it is reasonable to assume that teachers’ emotional intelligence affects teaching for creativity. However, existing research has failed to reach consistent conclusions about the relationship between teachers’ emotional intelligence and their creativity. Meanwhile, very few studies have explored why and how EFL teachers’ EI facilitates students’ creativity in the educational field (Carmeli et al., 2014). The relationship between teachers’ EI, WE and TfC is unclear as well. This can be seen as a gap in the literature that needs to be filled. In this sense, the purpose of this study was to examine the relationship between teachers’ EI and TfC and to further explore what role teachers’ WE plays in the relationship between EI and TfC.

### 1.1. The relationship between emotional intelligence and teaching for creativity

EI is the ability to detect feelings and emotions in oneself and others (Salovey and Mayer, 1997) and includes self-control, persistence, zeal, and the ability to self-motivate (Goleman, 1995). EI is a key research topic in management, education and psychology and has been found to be positively correlated with superior work performance in multiple work areas (Lavy and Eshet, 2018; Silva and Coelho, 2019). As two important qualities in the 21st century, a growing body of research has focused on the relationship between EI and creativity. Previous studies have found a strong relationship between EI and creativity (Xu et al., 2019; Tu et al., 2020) in various samples, including employees of travel agencies (Tsai and Lee, 2014), students (Chan, 2005; Tu et al., 2020), employees of software firms and service firms (Carmeli et al., 2014), salespersons (Lassk and Shepherd, 2013) and eldercare nurses (Toyama and Mauno, 2017). In addition, these studies have shown that people with high EI are able to maintain more positive emotions, mindfulness and sufficient intrinsic motivation at work, thus promoting creative self-efficacy and behavior (Oldham, 2003; Schaufeli et al., 2009; Zheng et al., 2022). In addition, Yang et al. (2021) conducted a qualitative analysis of 15,340 total sample dimensions in a correlation meta-analysis to determine the overall association between emotional intelligence and innovation. Through research on the relationship between emotional intelligence and creativity, it has been found that people with low EI are not good at handling unexpected events and tend to fall into negative emotions, while people with high EI are more likely to feel positive emotions and have the ability to transform negative emotions into change-oriented thinking processes. According to Fredrickson’s broaden-and-build theory, positive emotions expand our ability to think and act in the moment. When people have a positive mood, they are more open to information, more flexible and integrated in their thinking, and more innovative (Fredrickson, 1998). While emotional intelligence has led to better performance and human well-being in many areas, there is less research on teachers’ emotional intelligence and teaching for creativity.

In the field of teacher education research, there are individual studies on teachers’ emotional intelligence and creativity, but the results are controversial. Pirkhaefi and Rafieyan (2012) emphasized that teachers with high emotional intelligence and good mental health may enhance their pupils’ creativity level. In contrast, Ebrahimi et al. (2018) did not find a significant effect of emotional intelligence and creativity through an investigation of the relationship between emotional intelligence, emotions, and creativity among EFL teachers. Although few studies have explored the relationship between EI and TfC in the field of teacher research, the relationship between EI and other teachers’ teaching behavior characteristics has been extensively studied. High levels of EI support teacher wellbeing. Teachers who are better at regulating their emotions experience higher job satisfaction, positive impact, and are less likely to feel burned out (Latif et al., 2017; Turner and Stough, 2020). Specifically, Earlier research points out that teachers’ EI has a significant and optimistic association with several aspects of teaching, which comprise enjoyment in class (Frenzel et al., 2016; Kang, 2022), teacher efficacy beliefs (Uzuntiryaki-Kondakci et al., 2022), the relationship between teachers and students (Becker et al., 2014), burnout and engagement (Garrido and Pacheco, 2012; Yin et al., 2019).

Teacher emotional intelligence has a significant impact on the teaching and learning process, and teacher emotional intelligence has been shown to influence student learning behaviors, engagement, and academic performance (Corcoran and Tormey, 2013; Latif et al., 2017). Especially in second language teaching, the emotions of educators also have a significant impact on learners’ performance and success (Dewaele and MacIntyre, 2019; Wang et al., 2021; Shen, 2022). An increasing amount of research has begun to be directed toward the significance of teachers’ emotions and emotion regulation ability for language instruction and education (Dewaele and MacIntyre, 2019). Previous research has found that teachers’ EI is closely related to student learning, and teachers with high emotional intelligence are able to focus on the emotional aspects of learning and teaching exchanges (Mortiboys, 2005), which can directly create a positive atmosphere in the classroom to make the course more interesting and enjoyable (Miri and Pishghadam, 2021). It is an important condition for achieving TfC (Jeffrey and Craft, 2004; Sternberg, 2010). In addition, the feelings of educators in teaching and the emotional communication experience with learners, colleagues, managers and others directly affect the creativity of their teaching methods and then influence their learners’ presentation and success (Chang, 2013). Huang et al. (2021) has demonstrated through quantitative research that teacher enthusiasm as an important intrinsic factor has a positive impact on teaching for creativity. In pedagogy, researchers are increasingly finding that teachers’ EI is the third most important factor affecting teaching and learning, after subject expertise and learning and teaching methods (Mortiboys, 2005).

Some studies have explored the positive relationship between emotional intelligence and creativity in other domains, and others have confirmed the positive impact of teacher emotional intelligence on teacher well-being and teaching effectiveness. However, teachers’ EI and teaching for creativity is an issue worth exploring. This can be seen as a gap in the literature that needs to be filled urgently. In line with this, the following hypothesis has been formulated:

*H1*: Teachers’ emotional intelligence will positively affect teaching for creativity.

### 1.2. The mediating effect of teachers’ work engagement on the relationship between teachers’ EI and TfC

First, existing research has proven the relationship between EI and WE in many research areas. WE is considered a positive motivational structure for work, consisting of absorption, dedication and vigor (Schaufeli et al., 2002), which is an important topic of concern for positive psychology (Mills et al., 2013). Job demands-resources (JD-R) theory (Bakker and Demerouti, 2017) offers an explanatory model of the relationships among WE and its antecedents and consequences. Personal resources are positively correlated with WE (Bakker and Demerouti, 2017). As a typical personal resource, EI has been recognized as a positive predictor of work engagement (Pena et al., 2012; Zhu et al., 2015; Mérida-López and Extremera, 2020). In recent years, an increasing number of studies have shown that people with high EI are able to pay attention to emotions in their surroundings and develop strategies to manage and regulate their emotions and those of others, thus creating a positive work atmosphere and maintaining a positive emotional state, resulting in increased vigor, dedication and commitment at work (Extremera et al., 2012; Bakker et al., 2014; Fu et al., 2021). For teachers, EI is considered among the greatest job-related factors for their occupational health, well-being, and WE (Hakanen et al., 2006; Mérida-López et al., 2019). Teachers with high EI can better manage their emotions with others so that they can better control their work and reduce stress and burnout at work (Nikolaou and Tsaousis, 2002; Fu et al., 2021;). Meanwhile, the EI of teachers and students also affects students’ academic engagement and performance (Carmona-Halty et al., 2021). As a profession with high emotional demands, teachers’ EI and emotional labor directly affect their WE (Bakker and Bal, 2010). Overall, previous research has demonstrated that teachers’ WE is inseparable from their EI.

Second, research in other fields has concentrated on the mediating role of WE in the relationship between EI and creativity in various samples, such as employees (Carmeli et al., 2014) and eldercare nurses (Toyama and Mauno, 2017). Carmeli et al. (2014) surveyed the employees of three firms. The results of structural equation modeling (SEM) have indicated that employees with high EI show a high level of generosity, and generosity cultivates vigor at work, thereby promoting employees’ creativity. Toyama and Mauno (2017) also found that, in nurses, EI can enhance WE, which subsequently facilitates creativity. In general, there are studies and theories in other fields that have support that creativity is vulnerable to emotions and emotional intelligence.

Third, although little attention has been given to the impact of teachers’ work engagement on TfC in teacher education research, a direct relationship between the two can be hypothesized based on research on this issue in other fields and the theory of teachers’ positive emotion. Research in many fields has proven that a positive working state characterized by vigor, absorption and dedication can promote creativity (Schaufeli et al., 2009). For instance, Asif and his collaborator found that when employees become more engaged in their work, they generate creative ideas and show sensitivity to a problem (Asif et al., 2019). In the field of educational research, although it has not been proven by studies that WE has an effect on TfC, some studies have proven that some positive emotional experiences by teachers that are similar to WE, such as teachers’ enthusiasm (Frenzel et al., 2009; Huang et al., 2021) and enjoyment (Frenzel et al., 2016), are powerful predictors of TfC. As proposed by broaden-and-build theory, people with positive emotions are more willing to explore new strategies and learn more to expand their scope of cognition (Friedman and Förster, 2010) and enhance task engagement (De Dreu et al., 2008), which can enhance creativity. In the field of education, research on the relationship between emotional intelligence and creative teaching is relatively scarce, and the results of individual studies are not consistent. The relationship between teachers’ emotional intelligence and teaching for creativity and work engagement is also unclear. As Furnham (2016) pointed out, research on emotional intelligence and creativity is inadequate; therefore, there is a necessity to conduct this study.

As teaching is a profession with high emotional input, we believe that teachers’ EI has a stronger connection with WE and TfC. We investigated junior high school English teachers in Jilin Province, China, to investigate the relationships among teachers’ EI and WE and TfC. In this sense, this article seeks answers for the following hypotheses:

*H2*: Teachers’ emotional intelligence is positively correlated with their work engagement.

*H3*: Teachers’ work engagement mediates the relationship between emotional intelligence and teaching for creativity.

## 2. Materials and methods

### 2.1. Participants and procedure

Data were collected from 3,307 secondary school English teachers in Jilin Province, China. Jilin Province is located in Northeastern China. The educational level of secondary schools in Jilin Province was equivalent to the national average level. For example, the student-teacher ratio of Jilin in 2020 was 10.34, and the national average was 12.72 (China Education Statistical Yearbook, 2021; Jilin Statistical Yearbook, 2021). We distributed the questionnaires to teachers *via* the internet and explained that the study was designed to investigate the work of English teachers in secondary schools and asked for voluntary participation. Eventually, 3,307 questionnaires were collected. The sample comprised 252 males (7.5%) and 3,112 females (92.5%).

### 2.2. Measures

The original questionnaires used in this study were all in English. Therefore, we used a back translation method to translate the self-report questionnaires into Chinese for data collection (Brislin, 1980). Participants were asked to respond to these questionnaires on 5-point Likert scales (from 1 = totally disagree to 5 = totally agree), and higher scores indicate higher levels of variables.

#### 2.2.1. Teachers’ emotional intelligence

Teachers’ EI was measured using a 16-item scale developed by Wong and Law (2002). It measures four factors: self-emotion appraisal (SEA, four items, e.g., I really understand what I feel.), others’ emotion appraisal (OEA, four items, e.g., I have good understanding of the emotions of people around me.), use of emotion (UOE, four items, e.g., I always set goals for myself and then try my best to achieve them.), and regulation of emotion (ROE, four items, e.g., I have good control of my own emotions.). Cronbach’s alpha coefficient for EI was 0.86 in this study.

#### 2.2.2. Work engagement scale for teachers

The 17-item Utrecht Work Engagement Scale (Schaufeli et al., 2002) was used to measure teachers’ WE. This scale has three dimensions: vigor (six items; e.g., At my job I feel strong and vigorous.), dedication (five items; e.g., I am proud on the work that I do.), and absorption (six items; e.g., Time flies when I am working.). Cronbach’s alpha coefficients for the scales were 0.96 in this study.

#### 2.2.3. Teaching for creativity

A 9-item scale developed by Huang and colleagues (Huang and Lee, 2015; Huang, 2021) was used to measure teachers’ attitudes toward the implementation of TfC in their classrooms. TfC presupposes teachers’ pedagogical creativity, but its ultimate goal is to cultivate students’ creativity, which is a more complex kind of creativity (Jeffrey and Craft, 2004). This scale contains two dimensions: product-oriented and process-oriented. A sample scale item is “I inspire student creativity in my teaching.” Cronbach’s alpha coefficient for TfC was 0.91 in this study.

### 2.3. Data analysis

This quantitative study uses the structural equation model to verify the relationship between EI as the independent variable, WE as the intermediary variable and TfC as the dependent variable, and to verify whether the intermediary effect is significant. The data were analyzed with the IBM Statistics SPSS 24 program and IBM SPSS Amos 26 software. First, we used the IBM Statistics SPSS 24 program to describe the data and the correlation between the variables. Descriptive statistics analyzes also determined the means and standard deviations of these variables. Then, structural equation modeling (SEM) Amos 26 was conducted to examine between the components and examine multiple metrics of model fit. For the structural equation model, we used several statistics to determine the fit of each model, including the chi-square, goodness-of-fit index (GFI), adjusted goodness-of-fit index (AGFI), comparative fit index (CFI), and root-mean-square error of approximation (RMSEA). Finally, we used bootstrapping tests as estimators for testing mediation effects; zero was not straddled in the 95% confidence interval generated by the bias-corrected bootstrap method set to 5,000 reiterations for unstandardized and standardized estimates.

## 3. Results

### 3.1. Descriptive statistics

The means, standard deviations and correlations between variables are presented in Table 1. Before conducting a formal test of the hypothetical model, a test of the correlations among all variables was conducted to gain a preliminary understanding of the data. The results showed that the correlations for most of the variables were in the expected directions. Teachers’ EI was positively related to WE (*r* = 0.39, *p* < 0.01). TfC was positively correlated with WE (*r* = 0.41, *p* < 0.01) and teachers’ EI (*r* = 0.48, *p* < 0.01).

**Table 1:** Descriptive statistics and correlations between variables.

	Gender	Teaching length	EI	WE	TfC
Gender	-				
Teaching length	−0.110**	-			
EI	0.00	0.03	-		
WE	0.00	0.109**	0.385**	-	
TfC	−0.01	0.035*	0.483**	0.414**	-
Mean	-	16.69	3.70	3.82	4.08
SD	-	9.92	0.63	0.71	0.54

*p < 0.05; **p < 0.01; ***p < 0.001.

**Figure 1:**
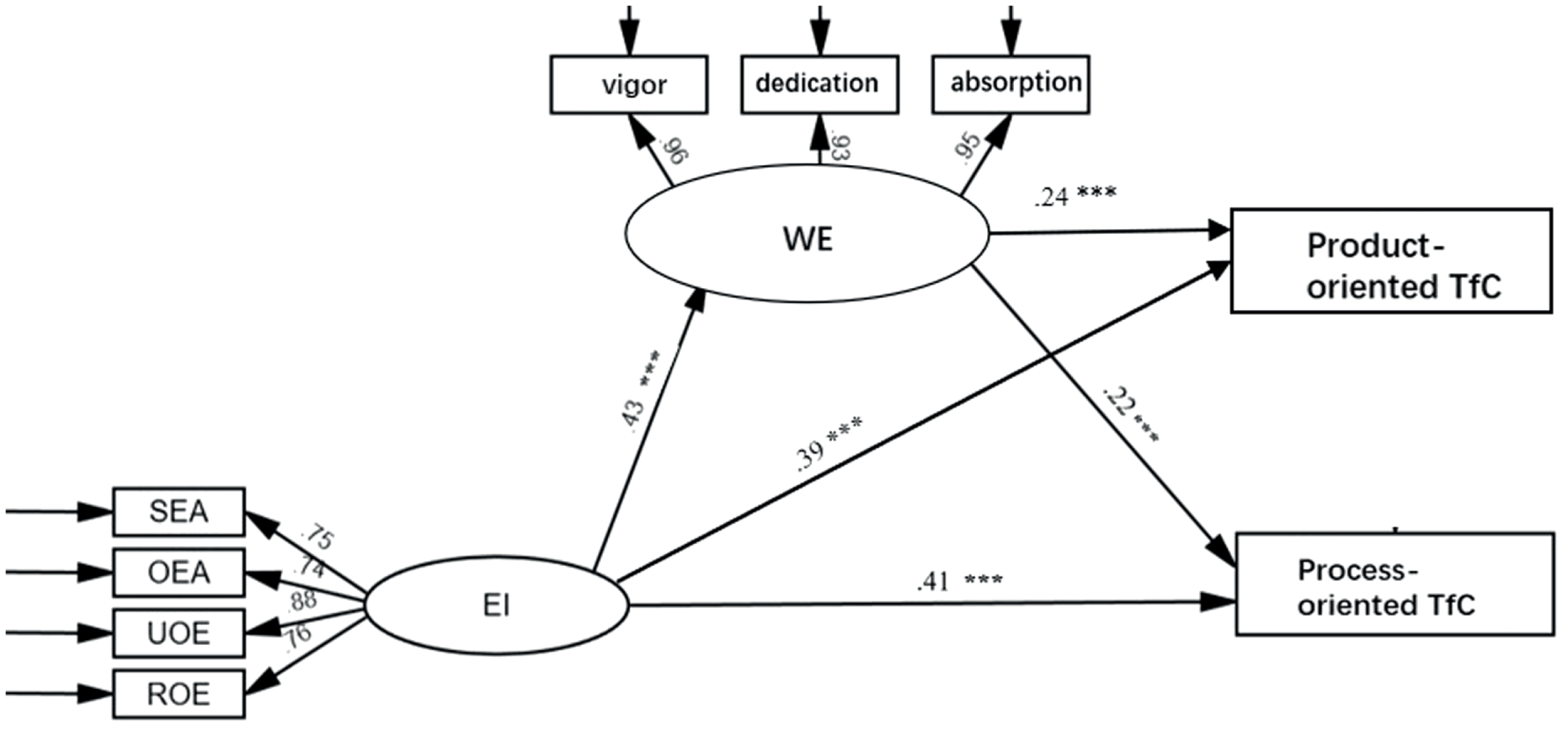
Structural model of the relationships among EI, WE and TfC. (Standardized regression weights are reported). *p < 0.05, **p < 0.01, ***p < 0.001.

To examine the link of teachers’ EI and WE with TfC, an SEM involving Teachers’ EI as a predictor, WE as a mediator and TfC as the outcome variable was tested. The structural model, with significant standardized path coefficients, is displayed in Figure 1. When product-oriented TfC was used as the outcome variable, the model fit the data well: CFI = 0.984, GFI = 0.973, TLI = 0.975, RMSEA = 0.074, SRMR = 0.020. When process-oriented TfC was used as the outcome variable, there was also a good model fit: CFI = 0.983, GFI = 0.972, TLI = 0.973, RMSEA = 0.076, SRMR = 0.020. From the results, we find that teachers’ EI (SEA, OEA, UOE, and ROE) directly affects teachers’ WE (vigor, dedication and absorption) and TfC. In particular, teachers’ EI significantly influences their WE (*B* = 0.43, *p* < 0.001), and it is significantly and positively correlated with product-oriented TfC (*B* = 0.39, *p* < 0.001) and process-oriented TfC (*B* = 0.41, *p* < 0.001). Meanwhile, teachers’ WE also directly influences TfC (product-oriented TfC *B* = 0.24, *p* < 0.001; process-oriented TfC *B* = 0.22, *p* < 0.001).

The test for intermediate effects is then performed by the bootstrap method. The 95% confidence interval for the bias-corrected bootstrap of the indirect effect was derived from 5,000 resamples. A mediated effect is considered significant if the interval does not straddle zero. Table 2 presents the bias-corrected 95% confidence interval of [0.068, 0.106] for the indirect effect of teachers’ EI on TfC (process-oriented) *via* WE, which did not contain zero. The bias-corrected 95% confidence interval of [0.077, 0.117] for the indirect effect of teachers’ EI on product-oriented TfC *via* WE also did not contain zero. This result suggests that teachers’ WE mediates the role of teachers’ EI and TfC.

**Table 2:** Bootstrap significance test of mediation effect (*n* = 3,307 bootstrap 5,000).

			Bias-corrected 95%CI			Percentile 95%CI		
The path	Effect of value	SE	Lower	Upper	*p*	Lower	Upper	*p*
Direct effect (EI → Product-oriented TfC)	0.374	0.026	0.322	0.425	0.000	0.322	0.425	0.000
Indirect effect (EI → WE → Product-oriented TfC)	0.097	0.010	0.077	0.117	0.000	0.078	0.118	0.000
Total effect (EI → WE→Product-oriented TfC)	0.471	0.023	0.426	0.517	0.000	0.426	0.516	0.000
Direct effect (EI → Process-oriented TfC)	0.374	0,025	0.323	0.421	0.001	0.323	0.422	0.000
Indirect effect (EI → WE→Process-oriented TfC)	0.087	0.010	0.068	0.106	0.002	0.069	0.107	0.000
Total effect (EI → WE → Process-oriented TfC)	0.461	0.022	0.418	0.503	0.001	0.419	0.504	0.000

## 4. Discussion

This quantitative study revealed the complex link between teachers’ WE, EI and creativity. Mediation analysis revealed that teachers’ emotional intelligence affected teaching for creativity directly and indirectly. The indirect link was related to teachers’ work engagement. Correlation analyzes confirmed positive correlations among EI, WE, and TfC. The results of this study show that EI positively predicts WE, which is consistent with previous findings (Constandin and Brate, 2015; Kabar and Gunes, 2017; Mérida-López and Extremera, 2020; Butakor et al., 2021; Fu et al., 2021; Sezen-Gultekin et al., 2021). Meanwhile, we found a statistically significant and positive relationship between TfC and teachers’ EI levels in the research. This conclusion has also been confirmed in studies with various samples, such as students (Chan, 2005), travel agents (Tsai and Lee, 2014), and eldercare nurses (Toyama and Mauno, 2017). Some research has suggested that high EI enables people to maintain a high level of positive emotions, thus promoting the continuous exploration of new ideas to enhance creativity (Baas et al., 2008; Hennessey and Amabile, 2010; Parke et al., 2015). This study also proves the mediating role of teachers’ WE in the relationship between EI and TfC.

First, the results suggest that EI and WE are positively associated, which is consistent with previous findings. As a positive affective–emotional sense of accomplishment (Schaufeli and Bakker, 2010), work engagement is a necessary positive emotion for teachers’ work. From the perspective of teachers’ internal emotional activities, we proposed that EI enables teachers to develop positive affective–emotional sense through emotion regulation ability—the ability to manage their own and others’ emotions (Mayer et al., 2008). This helps teachers sustain higher levels of positive affect (PA) or “a state of high energy, full concentration, and pleasurable engagement” (Watson et al., 1988, p.1063). From the external environment, we suggest that emotionally astute individuals can better evaluate and understand the emotions of others, such as students and colleagues (Carmona-Halty et al., 2021; Huang et al., 2021). High EI enables teachers to achieve dedication, generosity and other behaviors in the appropriate environment to cultivate high-quality interpersonal relationships and engender vigor, which also reinforces engagement in favor exchange.

Second, this study demonstrates the affirmative role of teachers’ EI on TfC, especially with process-oriented TfC. TfC requires teachers to provide students with opportunities to participate in creative activities and help students creatively apply knowledge to their lives (Jeffrey and Craft, 2004). TfC also involves helping students improve their creative learning ability. Several studies have suggested that emotions are bound up with learning (Rogers, 1983; Salovey and Mayer, 1997; Mortiboys, 2005). In the words of Guys Claxton, ‘learning itself is an intrinsically emotional business’ (Claxton, 1999, p. 15). Thus, the development of EI is a requirement for both teachers’ professional development and TfC. On the one hand, teachers’ EI is closely linked to their subject knowledge and teaching methods (Mortiboys, 2005). Teachers with high EI are better able to regulate their emotions and reflect and monitor their teaching behavior to develop their professional competencies, explore new teaching methods and learn more professional knowledge. As previous research has shown, constant reflection on experience and the constant exploration of new approaches lead to creative thinking and behavior (Hao et al., 2016; Thorley, 2018) and is strongly associated with process-oriented TfC in particular (Huang, 2021). High EI can also help people to be more flexible and adaptable in the face of unforeseen circumstances (Messmann and Mulder, 2015). For example, teachers with higher EI are able to be open and flexible in adjusting their teaching strategies in the face of students’ diverse, creative and unexpected opinions in the classroom. On the other hand, the ability of teachers with high EI to perceive and regulate the emotions of others facilitates their collaboration with stakeholders such as school administrators, colleagues, parents, and others (Fu et al., 2021) to build a creativity-supportive environment and obtain more external support for the TfC (Huang, 2021). In addition, teachers with high EI are better able to regulate their own emotions and those of their students, making it easier to build friendly, open, equal and trusting relationships with students. Yılmaz and Yılmaz (2022) demonstrated that a great sense of community enhances students’ emotional and behavioral engagement in learning, which also contributes to realizing TfC (Jeffrey and Craft, 2004; Hoy and Weinstein, 2013; Huang, 2021). Therefore, regardless of teachers’ personal resources or external environments, teachers’ EI is closely associated with TfC. Based on this, as emotional workers, teachers’ EI affects not only their professional development but also the core factor of TfC. Schools should actively adopt EI intervention strategies for teachers, which will help improve their EI and increase their positive emotions to encourage them to adopt more creative teaching strategies, build a creativity-friendly teaching atmosphere, and facilitate students’ innovation. Meanwhile, teacher educators can teach emotional regulation and creative teaching methods in teacher education courses and programs to cultivate preservice teachers with innovative spirit and positive emotions.

The results of this study indicate that teachers’ WE mediates the relationship between EI and TfC. In the field of management, previous studies have shown that EI is positively related to generosity and vigor, which in turn are positively associated with creativity (Carmeli et al., 2014). This conclusion was also confirmed in teacher research. Specifically, teachers with high EI are more involved in their work. Conversely, teachers who are energetic, focused, and committed at work are better at regulating their emotions at work. Affective information processing theory suggests that EI has two dimensions—emotion regulation and emotional facilitation—both of which can shape a positive emotion and can help teachers be more involved in their work. Emotional regulation enables teachers to maintain positive emotions in the face of work demands to produce good creative self-efficacy and create an energetic, fulfilling and focused work state. Meanwhile, teachers with high EI are better at regulating students’ emotions, thus contributing to increased emotional engagement, satisfaction and motivation, and promoting creativity in student learning (Yılmaz, 2022). Emotional facilitation enables people to use this positive state to transform creative ideas into creative behaviors and improve creativity at work (Parke et al., 2015). High WE makes teachers passionate about their work and more dedicated to teaching students with not only basic teaching methods but also innovative teaching methods and content to guide students to develop their own talents and creative abilities (Fu et al., 2021). Previous studies have found that as long as there is more job involvement input, teachers’ creative teaching performance is significantly improved. Because teachers become increasingly focused on their work, being proud of their work and immersing themselves in it will have a positive impact on TfC (Xiong et al., 2019). On the other hand, according to job demands-resources (JD-R) theory (Bakker and Demerouti, 2017), teachers with higher EI are able to better monitor and regulate the emotions of others, thus forming a good cooperative relationship with colleagues and having more opportunities to obtain work resources supported by the school. Schools should provide more opportunities for teachers to engage in peer-to-peer exchanges inside and outside of school and create a creativity-supportive teaching environment, which can strengthen the creativity of the team and individual. Previous research has shown that school management policies, such as performance-based accountability (PBA), and decision-making methods can affect teachers’ emotions (Tsang, 2019; Zhang and Tsang, 2021). Therefore, changing management styles and encouraging teachers to actively participate in decision-making about curriculum reform can keep teachers motivated and creative in their teaching. Meanwhile, a good colleague relationship and working environment will also encourage teachers to be more energetic, focused and dedicated to their teaching work and full of enthusiasm and pride for their work. Teachers who enjoy their work are more willing to explore novel pedagogies, remain open to the views of various students, and promote students’ innovation (Huang, 2021).

### 4.1. Limitations and future research

Undeniably, this study has some limitations. First, this study mainly used a cross-sectional research design, making it difficult to explore the causal relationships among variables, as a longitudinal research design will be conducted in the future. Second, the survey in this study was mainly in the form of a subjective report, thus generating common method bias (Podsakoff et al., 2003). Therefore, objective measures will be introduced in future studies to fully examine the relationships among variables using various methods. Third, the participants in the study were mainly from cities in Northeastern China, which may limit the generalizability of the findings, so the range of cities sampled will be expanded in future studies. Finally, this study’s assessment of TfC came from teachers’ self-reports, which may have affected the objectivity of its results. In future research, studies that focus on students’ creativity effectiveness and behavior could be conducted, and the results will be compared.

## 5. Conclusion

This study aimed to explore the influence of teachers’ emotional intelligence on teaching for creativity, and to explore the intermediary role of job involvement between them. The findings indicated that teachers’ EI plays a crucial role in teachers’ beliefs and behaviors regarding TfC. Additionally, the findings demonstrated the mediating role of WE between EI and TfC. This research makes a unique contribution to the literature by proving that teachers’ emotional intelligence and work engagement has a positive impact on the cultivation of students’ creativity. In this sense, interventions aimed at improving teachers’ EI and WE may be useful in developing TfC. The results of this study are expected to assist instructors and instructional designers to improve teachers’ positive emotions, enhance teachers’ well-being, promote better teaching performance, and enhance students’ creativity.

## Data availability statement

The raw data supporting the conclusions of this article will be made available by the authors, without undue reservation.

## Ethics statement

The studies involving human participants were reviewed and approved by the studies involving human participants were reviewed and approved by the Research Ethics Committee, Faculty of Education, Northeast Normal University. The patients/participants provided their written informed consent to participate in this study.

## Author contributions

HS generate ideas, design research plans, collect and analyze data, establish models, write papers, and be responsible for all work generated before and after publication. JZ generate ideas, provide resources, obtain funding, provide guidance, supervise, and revise paper critically. MX collected and analyzed the data, and revised the paper critically. MZ data analysis and revised paper critically. All authors contributed to the article and approved the submitted version.

## Funding

This work was supported by a grant from The Social Science Fund of Jilin Province (PhD and Youth Support Program): A study on the differentiation of Chinese “generation Z” subculture language (No. 2022C78).

## Conflict of interest

The authors declare that the research was conducted in the absence of any commercial or financial relationships that could be construed as a potential conflict of interest.

## Publisher’s note

All claims expressed in this article are solely those of the authors and do not necessarily represent those of their affiliated organizations, or those of the publisher, the editors and the reviewers. Any product that may be evaluated in this article, or claim that may be made by its manufacturer, is not guaranteed or endorsed by the publisher.

## References

[ref1] Asif M. Qing M. Hwang J. Shi H. (2019). Ethical leadership, affective commitment, work engagement, and creativity: testing a multiple mediation approach. Sustainability 11:4489. doi: 10.3390/su11164489

[ref2] Awwad A. A. (2022). The impact of EFL teachers’ emotional intelligence and teacher-related variables on self-reported EFL teaching practices. World J. Engl. Lang. 12:166. doi: 10.5430/wjel.v12n6p166

[ref3] Baas M. De Dreu C. K. Nijstad B. A. (2008). A meta-analysis of 25 years of mood-creativity research: hedonic tone, activation, or regulatory focus? Psychol. Bull. 134, 779–806. doi: 10.1037/a0012815 18954157

[ref4] Bakker A. B. Bal P. M. (2010). Weekly work engagement and performance: a study among starting teachers. J. Occup. Organ. Psychol. 83, 189–206. doi: 10.1348/096317909X402596

[ref5] Bakker A. B. Demerouti E. (2017). Job demands-resources theory: taking stock and looking forward. J. Occup. Health Psychol. 22, 273–285. doi: 10.1037/ocp0000056, PMID: 27732008

[ref6] Bakker A. B. Demerouti E. Sanz-Vergel A. I. (2014). Burnout and work engagement: the JD–R approach. Annu. Rev. Organ. Psychol. Organ. Behav. 1, 389–411. doi: 10.1146/annurev-orgpsych-031413-091235

[ref7] Becker E. S. Goetz T. Morger V. Ranellucci J. (2014). The importance of teachers’ emotions and instructional behavior for their students' emotions – an experience sampling analysis. Teach. Teach. Educ. 43, 15–26. doi: 10.1016/j.tate.2014.05.002

[ref8] Beghetto R. A. Kaufman J. C. Baer J. (2014). Teaching for Creativity in the Common Core Classroom. New York: Teachers College Press.

[ref9] Bereczki E. O. Kárpáti A. (2018). Teachers’ beliefs about creativity and its nurture: a systematic review of the recent research literature. Educ. Res. Rev. 23, 25–56. doi: 10.1016/j.edurev.2017.10.003

[ref10] Brislin R. W. (1980). “Translation and content analysis of oral and written material” in Handbook of Cross-Cultural Psychology: Methodology. eds. Triandis H. C. Berry J. W. (Boston: Allyn and Bacon), 389–444.

[ref11] Butakor P. K. Guo Q. Adebanji A. O. (2021). Using structural equation modeling to examine the relationship between Ghanaian teachers' emotional intelligence, job satisfaction, professional identity, and work engagement. Psychol. Sch. 58, 534–552. doi: 10.1002/pits.22462

[ref12] Carmeli A. McKay A. S. Kaufman J. C. (2014). Emotional intelligence and creativity: the mediating role of generosity and vigor. J. Creat. Behav. 48, 290–309. doi: 10.1002/jocb.53

[ref13] Carmona-Halty M. Salanova M. Llorens S. Schaufeli W. B. (2021). Linking positive emotions and academic performance: the mediated role of academic psychological capital and academic engagement. Curr. Psychol. 40, 2938–2947. doi: 10.1007/s12144-019-00227-8

[ref14] Chan D. W. (2005). Self-perceived creativity, family hardiness, and emotional intelligence of Chinese gifted students in Hong Kong. J. Second. Gift. Educ. 16, 47–56. doi: 10.4219/jsge-2005-471

[ref15] Chan S. Yuen M. (2014). Creativity beliefs, creative personality and creativity-fostering practices of gifted education teachers and regular class teachers in Hong Kong. Think. Skills Creat. 14, 109–118. doi: 10.1016/j.tsc.2014.10.003

[ref16] Chang M. L. (2013). Toward a theoretical model to understand teacher emotions and teacher burnout in the context of student misbehavior: appraisal, regulation and coping. Motiv. Emot. 37, 799–817. doi: 10.1007/s11031-012-9335-0

[ref17] China Education Statistical Yearbook. (2021). *China Education Statistical Yearbook.* Available at: https://www.yearbookchina.com/navibooklist-n3022021706-1.html (Accessed August 8, 2022).

[ref18] Claxton G. (1999). Wise up: The Challenge of Lifelong Learning. London: Bloomsbury.

[ref19] Collie R. J. Shapka J. D. Perry N. E. (2011). Predicting teacher commitment: the impact of school climate and social–emotional learning. Psychol. Sch. 48, 1034–1048. doi: 10.1002/pits.20611

[ref20] Constandin M. Brate A. (2015). “Teacher motivation and emotional intelligence in elementary and special school” in Advanced Research in Health, Education and Social Sciences: Towards a Better Practice. eds. Milcu M. Matos M. Vasilescu I. P. (Bucuresti: Editura Universitară), 205–209.

[ref21] Corcoran R. P. Tormey R. (2013). Does emotional intelligence predict student teachers' performance? Teach. Teach. Educ. 35, 34–42. doi: 10.1016/j.tate.2013.04.008

[ref22] De Dreu C. K. Baas M. Nijstad B. A. (2008). Hedonic tone and activation level in the mood-creativity link: toward a dual pathway to creativity model. J. Pers. Soc. Psychol. 94, 739–756. doi: 10.1037/0022-3514.94.5.739, PMID: 18444736

[ref23] Dewaele J. M. MacIntyre P. (2019). “The predictive power of multicultural personality traits, learner and teacher variables on foreign language enjoyment and anxiety” in Evidence-based second language pedagogy: A collection of instructed second language acquisition studies. eds. Sato M. Loewen S. (Abingdon, UK: Routledge), 263–286.

[ref24] Ebrahimi M. R. Heydarnejad T. Najjari H. (2018). The interplay among emotions, creativity and emotional intelligence: a case of Iranian EFL teachers. Transl. Stud. 6:10.

[ref25] Extremera N. Sánchez-García M. Durán M. A. Rey L. (2012). Examining the psychometric properties of the Utrecht work engagement scale in two Spanish multi-occupational samples. Int. J. Sel. Assess. 20, 105–110. doi: 10.1111/j.1468-2389.2012.00583.x

[ref26] Fredrickson B. L. (1998). What good are positive emotions? Rev. Gen. Psychol. 2, 300–319. doi: 10.1037/1089-2680.2.3.300, PMID: 21850154 PMC3156001

[ref27] Frenzel A. C. Goetz T. Lüdtke O. Pekrun R. Sutton R. E. (2009). Emotional transmission in the classroom: exploring the relationship between teacher and student enjoyment. J. Educ. Psychol. 101, 705–716. doi: 10.1037/a0014695

[ref28] Frenzel A. C. Pekrun R. Goetz T. Daniels L. M. Durksen T. L. Becker-Kurz B.. (2016). Measuring teachers’ enjoyment, anger, and anxiety: the teacher emotions scales (TES). Contemp. Educ. Psychol. 46, 148–163. doi: 10.1016/j.cedpsych.2016.05.003

[ref29] Friedman R. S. Förster J. (2010). Implicit affective cues and attentional tuning: an integrative review. Psychol. Bull. 136, 875–893. doi: 10.1037/a0020495, PMID: 20804240 PMC2933078

[ref30] Fu W. Wang C. Tang W. Lu S. Wang Y. (2021). Emotional intelligence and well-being of special education teachers in China: the mediating role of work-engagement. Front. Psychol. 12:696561. doi: 10.3389/fpsyg.2021.696561, PMID: 34526933 PMC8435597

[ref31] Furnham A. (2016). The relationship between cognitive ability. Psychology 7, 193–197. doi: 10.4236/psych.2016.72021

[ref32] Garrido M. P. Pacheco N. E. (2012). Perceived emotional intelligence in primary school teachers and its relationship with levels of burnout and engagement. Rev. Educ. (Madrid) 359, 604–627. doi: 10.4438/1988-592X-RE-2011-359-109

[ref33] Goleman D. (1995). Emotional Intelligence: Why it can matter more than IQ. New York: Bantam Books.

[ref34] Hakanen J. J. Bakker A. B. Schaufeli W. B. (2006). Burnout and work engagement among teachers. J. Sch. Psychol. 43, 495–513. doi: 10.1016/j.jsp.2005.11.001

[ref35] Hao N. Ku Y. Liu M. Hu Y. Bodner M. Grabner R. H.. (2016). Reflection enhances creativity: beneficial effects of idea evaluation on idea generation. Brain Cogn. 103, 30–37. doi: 10.1016/j.bandc.2016.01.005, PMID: 26808451

[ref36] Hennessey B. A. Amabile T. (2010). Creativity. Annu. Rev. Psychol. 61, 569–598. doi: 10.1146/annurev.psych.093008.100416 19575609

[ref37] Hoy A. W. Weinstein C. S. (2013). “Student and teacher perspectives on classroom management” in Handbook of classroom management. eds. Evertson C. M. Weinstein C. S. (London: Routledge), 191–230.

[ref38] Huang X. (2021). Striving for better teaching and student creativity development: linking informal workplace learning and teaching for creativity. Think. Skills Creat. 41:100889. doi: 10.1016/j.tsc.2021.100889

[ref39] Huang X. Chin-Hsi L. Mingyao S. Peng X. (2021). What drives teaching for creativity? Dynamic componential modelling of the school environment, teacher enthusiasm, and metacognition. Teach. Teach. Educ. 107:103491. doi: 10.1016/j.tate.2021.103491

[ref40] Huang X. H. Lee J. C. K. (2015). Disclosing Hong Kong teacher beliefs regarding creative teaching: five different perspectives. Think. Skills Creat. 15, 37–47. doi: 10.1016/j.tsc.2014.11.003

[ref41] Jeffrey B. Craft A. (2004). Teaching creatively and teaching for creativity: distinctions and relationships. Educ. Stud. 30, 77–87. doi: 10.1080/0305569032000159750

[ref42] Jilin Statistical Yearbook (2021). *Jilin Statistical Yearbook 2021*. Available at: http://tjj.jl.gov.cn/tjsj/tjnj/2021/ml/indexe.htm (Accessed August 8, 2022).

[ref43] Kabar M. Gunes D. Z. (2017). Emotional Intelligence as a Predictor of High School Teachers’ Work Engagement. In: *3rd International Conference on Lifelong Learning and Leadership for All (ICLEL)*. Portugal: ICLEL, pp. 976–983.

[ref44] Kang D. M. (2022). An elementary school EFL teacher’s emotional intelligence and emotional labor. J. Lang. Identity Educ. 21, 1–14. doi: 10.1080/15348458.2020.1777867

[ref45] Kaufman J. C. Beghetto R. A. (2009). Beyond big and little: the four C model of creativity. Rev. Gen. Psychol. 13, 1–12. doi: 10.1037/a0013688

[ref46] Lassk F. Shepherd C. (2013). Exploring the relationship between emotional intelligence and salesperson creativity. J. Pers. Sell. Sales Manag. 33, 25–37. doi: 10.2753/PSS0885-3134330103

[ref47] Latif H. Majoka M. I. Khan M. I. (2017). Emotional intelligence and job performance of high school female teachers. Pak. J. Psychol. Res. 32, 333–351.

[ref48] Lavy S. Eshet R. (2018). Spiral effects of teachers’ emotions and emotion regulation strategies: evidence from a daily diary study. Teach. Teach. Educ. 73, 151–161. doi: 10.1016/j.tate.2018.04.001

[ref49] Mayer J. D. Roberts R. D. Barsade S. G. (2008). Human abilities: emotional intelligence. Annu. Rev. Psychol. 59, 507–536. doi: 10.1146/annurev.psych.59.103006.093646 17937602

[ref50] Mérida-López S. Bakker A. B. Extremera N. (2019). How does emotional intelligence help teachers to stay engaged? Cross-validation of a moderated mediation model. Pers. Individ. Differ. 151:109393. doi: 10.1016/j.paid.2019.04.048

[ref51] Mérida-López S. Extremera N. (2020). The interplay of emotional intelligence abilities and work engagement on job and life satisfaction: which emotional abilities matter most for secondary-school teachers? Front. Psychol. 11:563634. doi: 10.3389/fpsyg.2020.563634, PMID: 33192836 PMC7606868

[ref52] Messmann G. Mulder R. H. (2015). Reflection as a facilitator of teachers’ innovative work behaviour. Int. J. Train. Dev. 19, 125–137. doi: 10.1111/ijtd.12052

[ref53] Mills M. J. Fleck C. R. Kozikowski A. (2013). Positive psychology at work: a conceptual review, state-of-practice assessment, and a look ahead. J. Posit. Psychol. 8, 153–164. doi: 10.1080/17439760.2013.776622

[ref54] Miri M. A. Pishghadam R. (2021). Toward an emotioncy based education: a systematic review of the literature. Front. Psychol. 12:727186. doi: 10.3389/fpsyg.2021.727186, PMID: 34421775 PMC8374051

[ref55] Mortiboys A. (2005). Teaching with Emotional Intelligence a Step-by-step Guide for Higher and Further Education Professionals. London, New York: Routledge.

[ref56] NACCCE (1999). All our Futures: Creativity, Culture and Education. London: DFEE.

[ref57] Nikolaou I. Tsaousis I. (2002). Emotional intelligence in the workplace: exploring its effects on occupational stress and organizational commitment. Int. J. Organ. Anal. 10, 327–342. doi: 10.1108/eb028956

[ref58] Oldham G. R. (2003). “Stimulating and supporting creativity in organizations” in Managing Knowledge for Sustained Competitive Advantage: Designing Strategies for Effective Human Resource Management. eds. Jackson S. E. DeNisi A. Hitt M. A. (San Francisco, CA: Jossey-Bass), 243–273.

[ref59] Parke M. R. Seo M. G. Sherf E. N. (2015). Regulating and facilitating: the role of emotional intelligence in maintaining and using positive affect for creativity. J. Appl. Psychol. 100, 917–934. doi: 10.1037/a0038452, PMID: 25528247

[ref60] Pena M. Rey L. Extremera N. (2012). Life satisfaction and engagement in elementary and primary educators: differences in emotional intelligence and gender. Rev. Psicodidáctica 17, 341–358. doi: 10.1387/RevPsicodidact.1220

[ref61] Pirkhaefi A. Rafieyan H. (2012). Investigation the relationship between emotional intelligence and mental health of primary school teachers with Pupils' creativity in Behshar city. Innov. Creat. Humanit. 4, 19–35.

[ref62] Podsakoff P. M. MacKenzie S. B. Lee J. Y. Podsakoff N. P. (2003). Common method biases in behavioral research: a critical review of the literature and recommended remedies. J. Appl. Psychol. 88, 879–903. doi: 10.1037/0021-9010.88.5.879, PMID: 14516251

[ref63] Rogers C. R. (1983). Freedom to Learn. Ohio: Merril.

[ref64] Rubenstein L. D. Ridgley L. M. Callan G. L. Karami S. Ehlinger J. (2018). How teachers perceive factors that influence creativity development: applying a social cognitive theory perspective. Teach. Teach. Educ. 70, 100–110. doi: 10.1016/j.tate.2017.11.012

[ref65] Salovey P. Mayer J. (1997). “What is emotional intelligence?” in Emotional Development and Emotional Intelligence: Implications for Educators. eds. Salovey P. Sluyter D. (New York: Basic Books), 3–31.

[ref66] Sawyer R. K. (2006). Educating for innovation. Think. Skills Creat. 1, 41–48. doi: 10.1016/j.tsc.2005.08.001

[ref67] Schaufeli W. B. Bakker A. B. (2010). “Defining and measuring work engagement: bringing clarity to the concept” in Work Engagement: A Handbook of Essential Theory and Research. eds. Bakker A. B. Leiter M. P. (London: Psychology Press), 10–24.

[ref68] Schaufeli W. B. Bakker A. B. Van Rhenen W. (2009). How changes in job demands and resources predict burnout, work engagement, and sickness absenteeism. J. Organ. Behav. 30, 893–917. doi: 10.1002/job.595

[ref69] Schaufeli W. B. Salanova M. González-romá V. Bakker A. B. (2002). The measurement of engagement and burnout: a two sample confirmatory factor analytic approach. J. Happiness Stud. 3, 71–92. doi: 10.1023/A:1015630930326

[ref70] Sezen-Gultekin G. Bayrakcı M. Limon İ. (2021). The mediating role of organizational commitment on the relationship between emotional labor and work engagement of teachers. Front. Psychol. 12:648404. doi: 10.3389/fpsyg.2021.648404, PMID: 34290645 PMC8287209

[ref71] Shen G. (2022). Anxiety, boredom, and burnout among EFL teachers: the mediating role of emotion regulation. Front. Psychol. 13:842920. doi: 10.3389/fpsyg.2022.842920, PMID: 35360565 PMC8960723

[ref72] Silva D. Coelho A. (2019). The impact of emotional intelligence on creativity, the mediating role of worker attitudes and the moderating effects of individual success. J. Manag. Organ. 25, 284–302. doi: 10.1017/jmo.2018.60

[ref73] Starko A. J. (2017). Creativity in the Classroom: Schools of Curious Delight. London: Routledge.

[ref74] Sternberg R. J. (2010). “Teaching for creativity” in Nurturing Creativity in the Classroom. eds. Beghetto R. A. Kaufman J. C. (Cambridge: Cambridge University Press), 394–414.

[ref75] Thorley M. (2018). The role of failure in developing creativity in professional music recording and production. Think. Skills Creat. 30, 160–170. doi: 10.1016/j.tsc.2018.05.002

[ref76] Toyama H. Mauno S. (2017). Associations of trait emotional intelligence with social support, work engagement, and creativity in Japanese eldercare nurses. Jpn. Psychol. Res. 59, 14–25. doi: 10.1111/jpr.12139

[ref77] Tsai C. T. Lee Y. J. (2014). Emotional intelligence and employee creativity in travel agencies. Curr. Issues Tour. 17, 862–871. doi: 10.1080/13683500.2013.859232

[ref78] Tsang K. K. (2019). Teachers’ Work and Emotions: A Sociological Analysis. London: Routledge.

[ref79] Tu C. Guo J. Hatcher R. C. Kaufman J. C. (2020). The relationship between emotional intelligence and domain-specific and domain-general creativity. J. Creat. Behav. 54, 337–349. doi: 10.1002/jocb.369

[ref80] Turner K. Stough C. (2020). Pre-service teachers and emotional intelligence: a scoping review. Aust. Educ. Res. 47, 283–305. doi: 10.1007/s13384-019-00352-0

[ref81] Uzuntiryaki-Kondakci E. Kirbulut Z. D. Sarici E. Oktay O. (2022). Emotion regulation as a mediator of the influence of science teacher emotions on teacher efficacy beliefs. Educ. Stud. 48, 583–601. doi: 10.1080/03055698.2020.1793300

[ref82] Wang Y. Derakhshan A. Zhang L. J. (2021). Researching and practicing positive psychology in second/foreign language learning and teaching: the past, current status and future directions. Front. Psychol. 12:731721. doi: 10.3389/fpsyg.2021.731721, PMID: 34489835 PMC8417049

[ref83] Watson D. Clark L. A. Tellegen A. (1988). Development and validation of brief measures of positive and negative affect: the PANAS scales. J. Pers. Soc. Psychol. 54, 1063–1070. doi: 10.1037/0022-3514.54.6.1063, PMID: 3397865

[ref84] Wong C. S. Law K. S. (2002). The effects of leader and follower emotional intelligence on performance and attitude: an exploratory study. Leadersh. Q. 13, 243–274. doi: 10.1016/S1048-9843(02)00099-1

[ref85] Xiong Y. Sun X. Y. Liu X. Q. Wang P. Zheng B. (2019). The influence of self-efficacy and work input on physical education teachers’ creative teaching. Front. Psychol. 10:2856. doi: 10.3389/fpsyg.2019.02856, PMID: 31993003 PMC6964797

[ref86] Xu X. Liu W. Pang W. (2019). Are emotionally intelligent people more creative? A meta-analysis of the emotional intelligence–creativity link. Sustainability 11:6123. doi: 10.3390/su11216123

[ref87] Yang R. Díaz V. G. Hsu C. H. (2021). Use of emotional intelligence to promote innovation among employees in the work environment through qualitative and quantitative analysis. Aggress. Violent Behav. 1:101589. doi: 10.1016/j.avb.2021.101589

[ref88] Yılmaz F. G. K. (2022). An investigation into the role of course satisfaction on students’ engagement and motivation in a mobile-assisted learning management system flipped classroom. Technol. Pedagog. Educ. 31, 15–34. doi: 10.1080/1475939X.2021.1940257

[ref89] Yılmaz F. G. K. Yılmaz R. (2022). Exploring the role of sociability, sense of community and course satisfaction on students’ engagement in flipped classroom supported by Facebook groups. J. Comput. Educ. doi: 10.1007/s40692-022-00226-y

[ref90] Yin H. Huang S. Chen G. (2019). The relationships between teachers’ emotional labor and their burnout and satisfaction: a meta-analytic review. Educ. Res. Rev. 28:100283. doi: 10.1016/j.edurev.2019.100283

[ref91] Zhang Y. Tsang K. K. (2021). Performance-based accountability and teacher emotions: role of Zhongyong thinking. Front. Psychol. 12:612206. doi: 10.3389/fpsyg.2021.612206, PMID: 33927665 PMC8076591

[ref92] Zheng X. Ni D. Liu X. Liang L. H. (2022). Workplace mindfulness: multidimensional model, scale development and validation. J. Bus. Psychol. doi: 10.1007/s10869-022-09814-2

[ref93] Zhu Y. Liu C. Guo B. Zhao L. Lou F. (2015). The impact of emotional intelligence on work engagement of registered nurses: the mediating role of organizational justice. J. Clin. Nurs. 24, 2115–2124. doi: 10.1111/jocn.12807, PMID: 25894887

